# Effects of Dietary *Bacillus* and Non-starch Polysaccharase on the Intestinal Microbiota and the Associated Changes on the Growth Performance, Intestinal Morphology, and Serum Antioxidant Profiles in Ducks

**DOI:** 10.3389/fmicb.2021.786121

**Published:** 2021-12-08

**Authors:** Simin Peng, Xin Wang, Yuyu Wang, Tuo Lv, Haohan Zhao, Yanzhou Wang, Siyuan Zhu, Huajiao Qiu, Jianguo Zeng, Qiuzhong Dai, Qian Lin

**Affiliations:** ^1^Institute of Bast Fiber Crops, Chinese Academy of Agricultural Sciences, Changsha, China; ^2^Hunan Institute of Animal and Veterinary Science, Changsha, China; ^3^College of Veterinary Medicine, Hunan Agricultural University, Changsha, China

**Keywords:** gut microbiota, *Bacillus*, non-starch polysaccharidase, growth performance, serum antioxidant profiles, intestinal morphology, duck

## Abstract

Given the desirable results of using probiotics and enzyme preparations as feed supplements in poultry health, here, the effects of *Bacillus* and Non-starch Polysaccharase (NSPase) on the growth performance, serum antioxidant profiles, and gut microbial communities of early stage ducks is investigated. A total of 400 *Zhijiang* ducks (of similar body weight and 1 day age) was selected and randomly divided into four groups. The feeding period was 28 days. Each group contained 10 replicates of 10 birds. Control group (I) was fed with basal diet, while treatment groups II to IV were fed, respectively, with 150 mg/kg NSPases, 25 mg/kg *Bacillus* probiotics, and 150 mg/kg NSPases + 25 mg/kg *Bacillus* probiotics in their basal diet. The results demonstrated that dietary *Bacillus* (25 mg/kg) increased average final weight, average daily gain (ADG), and decreased the malonaldehyde (MDA) in birds (*P* < 0.05). Dietary *Bacillus* (25 mg/kg) and NSPases + *Bacillus* (150 mg/kg + 25 mg/kg) presented much higher glutathione (GSH) and activities of superoxide dismutase (SOD) in birds (*P* < 0.05). Additionally, as revealed by β-diversity indices and analysis of similarities, dietary NSPases + *Bacillus* could affect the ileum microbial abundances and diversities at the genera level (*P* < 0.05), but it had no effect on the caecal microbiota. Also, 16S rRNA sequencing revealed that dietary *Bacillus* and NSPases + *Bacillus* increased the populations of *Ruminococcaceae* genera in the cecum (*P* < 0.05), and *S24-7_group* and *Lactobacillus* genera in the ileum (*P* < 0.05). However, dietary NSPases and *Bacillus* alone and in combination could significantly decrease the content of *Bacteroides* in the ileum (*P* < 0.05). According to Spearman correlation analysis, 7 ilea bacterial microbiomes (*S24-7 group, Lactobacillus, Subgroup 2, Subgroup 1, Kitasatospora, Candidatus Solibacter, and Akkermansia*) were positively correlated with SOD (*P* < 0.05). In conclusion, *Bacillus* (25 mg/kg) and NSPases (150 mg/kg) included in the diet could efficiently enhance the growth performance by altered gut microbiota composition at the genera level and antioxidant indices of ducks.

## Introduction

Due to the antibiotic resistance of bacteria being on the rise (Guo et al., 2016) and the increasing consumer concern regarding poultry products, antibiotic-free flocks (Gadde et al., 2017), and environmental sustainability (Xing et al., 2015), a prohibition on antibiotics had been imposed in Europe (Stolker et al., 2007), South Korea (Kumar et al., 2012), and China (Hu and Cowling, 2020) since 2006, 2012, and 2020, respectively. Hence, replacing antibiotics with alternative products (Han et al., 2017) has become a hot research topic in recent years. Probiotics and enzymes are attracting much attention as important alternatives to antibiotics.

*Bacillus* is widely used in poultry diets as a type of probiotic, which improves the productive performance of poultry (Naumova et al., 2021) by producing naturally synthesized antimicrobial peptides, maintaining microbial flora balance in the intestie, accelerating the increase of beneficial microbiota along the gastrointestinal tract, and adjusting the immunological function and gut morphology (Sen et al., 2012; Grant et al., 2018). Many findings confirmed the significant improvement of growth performance, immune response, cecal microbial population, and intestinal morphology of weanling pig (Lee et al., 2014), Cherry Valley ducks (Guo et al., 2016), and Ross broiler chicks (Sen et al., 2012) with Bacillus-based diets. In addition, lacking enough enzymes to fully digest fiber, birds rely on exogenous enzymes in their diets to improve the fiber digestion process (Alagawany et al., 2018). As one of the most important exogenous enzymes, non-starch polysaccharidase (NSPase) is commonly used in poultry diets and plays many essential roles such as breaking down the non-starch polysaccharides, releasing nutrients encapsulated by the cell wall, reducing intestinal viscosity, improving animals’ utilization of nutrients, and affecting the composition and metabolic potential of bacterial populations (Kiarie et al., 2013; Ravindran, 2013; Amerah, 2015). Ao et al. (2010) and Coppedge et al. (2012) reported the supplementation of NSPase could improve the growth performance of market broilers and pigs and be used as a strategy to degrade antinutritional compounds so as to reduce dietary energy levels and costs.

Both *Bacillus* and NSPase can improve the productive performance of poultry, and are able to directly or indirectly affect the intestinal flora. However, it is not clear whether there is a synergistic effect between them. A few studies demonstrated that there was a certain synergistic effect on improving intestinal health, promoting nutrient digestion and growth in broiler chickens, when both *Bacillus* and NSPase were used as a supplement in the feed (Lin Q. et al., 2012; Wealleans et al., 2017). However, no such study has been conducted in ducks. In the present study, we aimed to study the effects of *Bacillus* and NSPase on the growth performance, serum antioxidant profiles, intestine morphology, and intestinal microbiota composition in ducks.

## Materials and Methods

All the experimental procedures of this study were approved by the Animal Care and Use Committee of the Institute of Bast Fiber Crops, Chinese Academy of Agricultural Sciences.

### Experimental Design, Diets, and Birds

*Zhijiang* duck, an important indigenous bird breed in Southern China, is characterized by rapid growth, strong disease resistance, unique flavor, and delicious meat. Usually, they are sold as products in the marketplace at the age of 56 days, often with an average body weight of around 2,650 g for a single duck. From the age of 1–28 days, *Zhijiang* duck are at the critical stage of intestinal development and maximum growth. Therefore, a total of 400 female *Zhijiang* ducks (aged 1 day and free of infectious disease) were obtained from Hunan Hexiang Duck Industrial Co., Ltd. They were then transferred into the laboratory of the Bast Fiber Crops Institute, Chinese Academy of Agricultural Sciences for a feeding period of 28 days. Feed and water were provided *ad libitum* during the whole trial period. Each *Zhijiang* duck was weighed at the beginning to obtain the average initial weight, in order to divide them into four groups without significant difference among groups. Each group (100 *Zhijiang* ducks) was further subdivided into 10 cages (10 ducks/cage), and the dimension of each cage was 150 cm × 150 cm. Group I received a basal diet (BD), Group II received BD supplemented with 150 mg/kg NSPases (Manufactured by Shanghai CJYOUTELL Biotechnology Co., Ltd., China); the main components of this NSPases are cellulase (≥ 2,000 IU/g) and xylanase (≥ 30,000 IU/g). Group II received BD supplemented with 25 mg/kg *Bacillus* probiotics; the main components being *Bacillus subtilis* and *Bacillus lichenif or mi s*, 5 × 10^12^CFU/g, manufactured by Wuhan Xiongfeng Technology Co., Ltd., China. Group IV received BD supplemented with 150 mg/kg NSPases + 25 mg/kg *Bacillus* probiotics. The BD was prepared in accordance with the Nutrient Requirements of Ducks (National Research Council, 1994) and the Nutrient Requirements of Meat-type Duck (China, NY/T 2122-2012) (Ministry of Agriculture of the People’s Republic of China, 2012; Table 1).

**TABLE 1:** Composition and nutrient levels of basal diets (air-dry basis,%).

Item	Ingredients	Item	Nutrient levels^b^
Corn	46.95	ME/(Mcal/kg)	2.83
Soybean meal	25.30	CP	17.37
Rice bran	9.00	Calcium	0.90
Barley	14.52	Total P	0.68
Limestone	1.53	Available P	0.36
CaHPO_4_⋅2H_2_O	1.20	NaCl	0.34
NaCl	0.30	Lys	1.00
98.5% *DL*- Met	0.11	Met	0.39
78% *L*- Lys	0.09	Met + Cys	0.68
1% Premix^a^	1.00	CF	3.34
Total	100.00		

*^a^The premix provided the following (per kilogram of complete diet) micronutrients: VA 12 000 IU, VD_3_ 2 500 IU, VE 20 mg, VK_3_ 3 mg, VB_1_ 3 mg, VB_2_ 8 mg, VB_6_ 7 mg, VB_12_ 0.03 mg, D-pantothenic acid 20 mg, nicotinic acid 50 mg, biotin 0.1 mg, folic acid 1.5 mg, Cu (as copper sulfate) 9 mg, Zn (as zinc sulfate) 110 mg, Fe (as ferrous sulfate) 100 mg, Mn (as manganese sulfate) 100 mg, Se (as sodium selenite) 0.16 mg, I (as potassium iodide) 0.6 mg. ^b^ Nutrient levels are calculated values.*

### Growth Performance

Birds were weighed at the beginning (day 1) and the end (day 28) of the trial for calculation of growth performance. Average daily weight gain (ADG), average daily feed intake (ADFI), and feed/gain ratio (F/G) were calculated according to the data from each cage for the whole experimental period. The birds were fasted for 12 h before weighting and sampling.

### Sample Collection

At the end of the trail, one bird per cage with a live weight close to the mean (10 birds per group) were selected for sampling. Blood samples from the wing vein were collected into vacuum blood collection tubes and were centrifuged at 3,000 × g for 10 min to collect the serums. Birds were slaughtered by exsanguination from the jugular vein. Samples of the small intestine were immediately removed and then divided into three parts: Duodenum, jejunum, and ileum. Intestinal tissue samples were cut from the medial of the duodenum, jejunum, and ileum with a segment of 1.5 cm, and lightly flushed using physiological saline (154 mmol/L) and drained on filter paper. Then these fresh samples were fixed into 10% neutral buffered formalin for further analysis of intestinal mucosal morphology (Watkins et al., 2004; Applegate et al., 2005). Samples of chyme in each duck’s ileum and cecum were collected separately into 2 mL EP tubes and flash-frozen using liquid N_2_ and stored at –80°C until analysis.

### Serum Antioxidant Capacity

Samples of serum were analyzed for malonaldehyde (MDA), antioxidant biomarkers including glutathione (GSH), activities of superoxide dismutase (SOD),glutathione peroxidase (GSH-Px), total antioxidant capacity (T-AOC), and Catalase (CAT) were determined by the commercial assay kits (Nanjing Jiancheng Bioengineering Institute, China) with an automated fluorescence instrument (MultiskanM™ SkyHigh, Thermo Fisher Scientific, Waltham, MA, United States).

### Measurement of Intestinal Mucosal Morphology

The intestinal morphological measurement of the duodenum, jejunum, and ileum was based on the method reported in our previous research (Lin Q. et al., 2017). Briefly, 1.5 cm-intestinal tissue samples of the duodenum, jejunum, and ileum were fixed and embedded in paraffin, sectioned at a thickness of 5 or 6 μm using a microtome (RM-2235, Leica microsystems AG., Hessen, Germany), then mounted on glass slides and subsequently stained with hematoxylin and eosin (HE staining). We observed the finished slides and chose the typical microscopic fields to take photos of under an Olympus Van-Ox S microscope (Opelco, Washington, DC). Visual measurements of the villus height, crypt depth, and intestinal wall thickness from each slide were made on 10 readings at 40 × and 100 × magnifications using an image analysis system (Image-Pro, Media Cybernetics, Inc., Silver Springs, MD, United States). Then the ratios of villus height to crypt depth (V/C) can be calculated.

### Gut Microbiota Composition by 16S rRNA Gene Sequencing

The genomic DNA of the ileum and cecum microbial community were extracted according to the manufacturer’s instructions by the E.Z.N.A. DNA kits (Omega Bio-tech, Norcross, GA, United States). Ten ileum/cecum digesta samples were mixed in pairs for the 16S rDNA sequence determination. The 16S rRNA gene V3 + V4 regions of the bacteria were amplified with the primer pairs 806R (5′-GGACTACHVGGGTWTCTAAT-3′) and 338F (5′-ACTCCTACGGGAGGCAGCAG-3′) combined with adapter sequences and barcode sequences. Next, the DNA was purified by AxyPrep DNA Gel Extraction Kits (Axygen Bioscience, Union City, CA, United States). Finally, the sequencing of 16S rRNA gene was performed on an Illumina HiSeq 2500 platform (Illumina, San Diego, CA, United States) with purified amplicons were paired.

Reads with complete (length > 300 bp) sequence barcodes were screened for the following analysis. Obtained sequences of samples were filtered by using QIIME (version 1.17) software and the number of operational taxonomic units (OTUs) with a cut of 97% sequence similarity were determined by using UPARSE (version 7.1). Each OTU represented sequence was analyzed by Ramer-Douglas-Peucker (RDP) Classifier version 2.2 via the 16S rRNA database.

The alpha diversities of the ileal and cecal microbiota, including abundance-based coverage estimator (ACE), Chao1 estimator, Simpson and Shannon diversity index were calculated to investigate the richness and diversity of the community, respectively. Principal coordinate analysis (PCoA), and Non-MetricMulti- Dimensional Scaling (NMDS) were calculated to evaluate the difference of the microbial community based on the Bray-Curtis dissimilarity matrix. The analysis procedures of ileal and cecal microbiota were processed on the free online platform of BMK Cloud Platform (BioMarker Technologies Co., Ltd., Beijing, China).

### Statistical Analysis

One-way ANOVA model was performed to identify significant differences in growth performance, serum antioxidant profiles, intestinal mucosal morphology, and richness and community diversity of bacteria. All the results were presented as means plus standard errors of the means (SEM). Differences between means of all groups were considered significant at *P*-value less than 0.05. The analysis of statistical comparison was conducted on the basis of Student’s *t*-test to declare the difference of the relative abundance of the ileal and cecal microbiota between two groups. Spearman correlation analysis was performed to identify the relationship between ileum and cecum microbial community and measured parameters. Heatmap was constructed using Prism 9.0 (GraphPad Software, San Diego, CA). All the statistical analysis was done with SPSS 19.0 (IBM, Armonk, New York).

## Results

### Growth Performance

Growth performances are shown in Table 2. Compared to the control group I, the values of average final weight and ADG showed a significant increase in Group III, whereas Group II and Group IV exhibited an increasing trend with no significant difference (*P* > 0.05). The ADFI and F/G of ducks among any groups did not change significantly (*P* > 0.05) during the entire experimental period.

**TABLE 2:** Effects of each treatment^1^ on growth performance of Zhijiang ducks^2^ (1 28 days).

Item	Group I	Group II	Group III	Group IV	SEM	*P*-value
Average initial weight, g	45.72	45.82	45.84	45.80	0.048	0.835
Average final weight, g	1323.00^b^	1343.19^a,b^	1369.58^a^	1355.28^a,b^	6.161	0.046
ADG, g	47.31^b^	48.05^a,b^	49.03^a^	48.50^a,b^	0.228	0.046
ADFI, g	99.87	99.95	98.62	100.31	0.918	0.929
F/G	2.11	2.08	2.01	2.07	0.020	0.344

*ADG, average daily weight gain; ADFI, average daily feed intake; F/G, feed to gain ratio.*

*^1^Group I (control group), Group II (150 mg/kg NSPases in basal diet), Group III (25 mg/kg Bacillus probiotics in basal diet), Group IV (150 mg/kg NSPases + 25 mg/kg Bacillus probiotics in basal diet).*

*^2^Data is the mean of 10 replicates per treatment.*

*^a–b^Different superscript letters in the same row indicate significant differences at P < 0.05.*

### Serum Antioxidant Profiles

Six serum antioxidant indicators were presented in Table 3. GSH-Px (*P* = 0.878), T-AOC (*P* = 0.411) and CAT (*P* = 0.282) in different treatment group showed similar levels. But significant differences were told among groups in the serum levels of GSH, SOD, and MDA (*P* < 0.01). Compared to Group I, Groups III, and IV showed a significant increase in the serum level of SOD and GSH (*P* < 0.01). In contrast, the level of MDA showed a significant decrease in Groups II, III, and IV compared to that of Group I.

**TABLE 3:** Effects of each treatment^1^ on antioxidative parameters of Zhijiang ducks^2^ (1 28 days).

Item	Group I	Group II	Group III	Group IV	SEM	*P*-value
MDA, nmol/mL	7.00^a^	5.39^b^	3.65^c^	5.76^b^	0.306	<0.01
GSH, μmol/L	16.46^b^	17.12^b^	21.08^a^	19.81^a^	0.494	<0.01
SOD, U/mL	65.32^b^	68.83^b^	74.31^a^	76.31^a^	1.164	<0.01
GSH-Px, U/mL	193.97	189.16	187.39	188.28	2.908	0.878
T-AOC, mmol/mL	0.86	0.97	0.93	1.01	0.032	0.411
CAT, U/mL	1.03	0.92	0.96	0.87	0.029	0.282

*MDA, malonaldehyde; GSH, glutathione; SOD, superoxide dismutase; GSH-Px, glutathione peroxidase; T-AOC, total antioxidant capacity; CAT, Catalase.*

*^1^Group I (control group), Group II (150 mg/kg NSPases in basal diet), Group III (25 mg/kg Bacillus probiotics in basal diet), Group IV (150 mg/kg NSPases + 25 mg/kg Bacillus probiotics in basal diet).*

*^2^Data is the mean of 10 replicates per treatment.*

*^a–c^Different superscript letters in the same row indicate significant differences at P < 0.05.*

### Intestinal Mucosal Morphology

According to Table 4, no significant difference (*P* > 0.05) was observed on any intestinal morphology parameters of all groups. But for the duodenum, jejunum, and ileum, an increasing trend in villus height and V/C, and a decreasing trend in Crypt depth has been shown in Groups II, III, or IV when compared to Group I.

**TABLE 4:** Effect of each treatment^a^ on intestinal morphology of *Zhijiang* ducks^b^ (1 28 days).

Item	Group I	Group II	Group III	Group IV	SEM	*P*-value
**Duodenum**						
Villus height, μm	577.45	590.31	598.00	610.14	12.455	0.835
Crypt depth, μm	130.20	122.07	121.67	129.11	2.376	0.452
V/C	4.45	4.86	4.98	4.77	0.113	0.394
Intestinal wall thickness, μm	193.62	185.93	188.24	196.25	3.886	0.785
**Jejunum**						
Villus height, μm	582.71	586.32	606.55	602.88	11.677	0.866
Crypt depth, μm	129.62	122.12	117.19	125.40	2.392	0.314
V/C	4.53	4.88	5.23	4.85	0.124	0.256
Intestinal wall thickness, μm	192.18	190.62	176.98	184.00	3.161	0.313
**Ileum**						
Villus height, μm	544.05	554.70	562.82	537.81	8.685	0.761
Crypt depth, μm	123.71	117.50	121.86	120.09	2.201	0.794
V/C	4.42	4.76	4.65	4.51	0.073	0.356
Intestinal wall thickness, μm	192.30	182.38	183.14	182.94	3.292	0.684

*V/C, the ratios of villus height to crypt depth.*

*^a^Group I (control group), Group II (150 mg/kg NSPases in basal diet), Group III (25 mg/kg Bacillus probiotics in basal diet), Group IV (150 mg/kg NSPases + 25 mg/kg Bacillus probiotics in basal diet).*

*^b^Data is the mean of 10 replicates per treatment.*

### Modulation of Intestinal Microbiota

**FIGURE 1:**
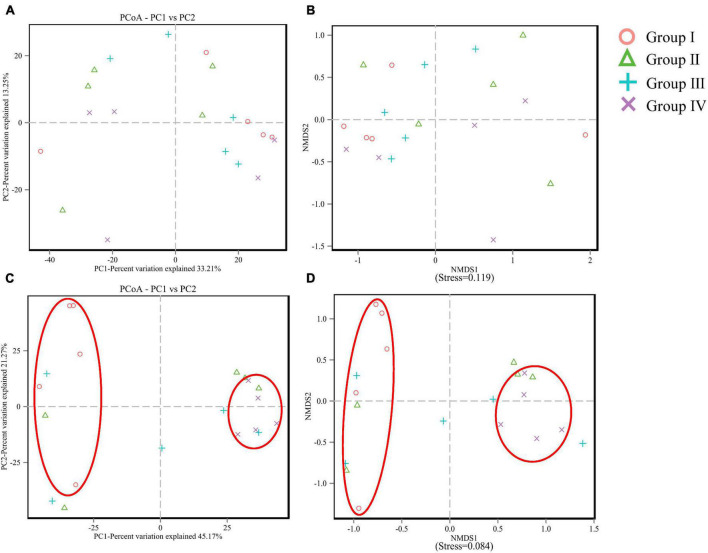
PCoA and NMDS analysis of cecum **(A,B)** and ileum **(C,D)** microbial community compositions based on information of operational taxonomic units (OTU).

Shannon, Simpson, ACE, and Chao, are important indexes of the α diversity of a bacterial community. The results (as shown in Table 5) were obtained through the 16S rRNA sequencing. For ileum, the values of ACE (*P* = 0.007) and Chao (*P* = 0.006) showed a significant increasing trend in Group IV, and they also increased in Group II and Group III. But instead, the α diversity parameters of the cecum showed no change (*P* > 0.05) among groups. PCoA and NMDS revealed the variation between microbiome profiles based upon Bray-Curtis dissimilarity. For cecum, PCoA plot (Figure 1A) and NMDS plot (Figure 1B) revealed no differences in microorganism distributions between the four groups at the genera level. However, in the ileum, samples from Group I and Group II were clearly distributed in both clusters, on the left and right parts of both PC1 (Figure 1C) and NMDS1 (Figure 1D), respectively.

**TABLE 5:** α diversity of cecum and ileum microbial community in different groups^1,2^.

Item	Group I	Group II	Group III	Group IV	SEM	*P*-value
**Cecum**						
Shannon	3.00	3.61	3.47	3.19	0.135	0.404
Simpson	0.208	0.089	0.114	0.147	0.023	0.319
ACE	294.40	311.18	304.62	305.61	4.449	0.639
Chao	299.04	313.05	304.77	309.10	4.604	0.766
**Ileum**						
Shannon	3.15926	4.22804	3.84302	5.84306	0.391	0.081
Simpson	0.13528	0.1852	0.16514	0.01094	0.043	0.508
ACE	460.473^b^	667.262^b^	735.252^a,b^	975.853^a^	58.653	0.007
Chao	441.658^c^	674.64^b,c^	740.653^a,b^	982.825^a^	60.437	0.006

*^1^Group I (control group), Group II (150 mg/kg NSPases in basal diet), Group III (25 mg/kg Bacillus probiotics in basal diet), Group IV (150 mg/kg NSPases + 25 mg/kg Bacillus probiotics in basal diet).*

*^2^Data is the mean of 5 replicates per treatment.*

*^a–c^Different superscript letters in the same row indicate significant differences at P < 0.05.*

**FIGURE 2:**
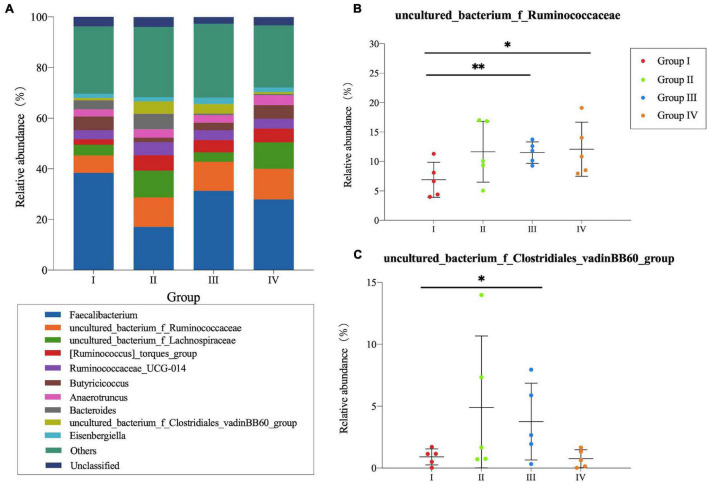
Community structure of bacteria at genus level in the cecum of *Zhijiang* ducks. Histogram **(A)** of the top 10 genera in each group. Significant difference relative abundance of *Ruminococcaceae* **(B)** and *vadinBB60_group* **(C)** at the genera level. (*P < 0.05 and ^**^P < 0.01 compared with control group).

In cecum, microbial communities among different groups at the genus level are shown in Figure 2A. *Faecalibacterium*, *Ruminococcaceae*, *Lachnospiraceae*, *Torques group*, and *UCG-014* were the dominant microbes and occupied approximately 50% of the total genera. *Faecalibacterium* was detected in the highest abundance in Group I, whereas the *Ruminococcaceae* and *Torques group* showed lower abundance than other groups. As for *Lachnospiraceae*, higher abundance was observed in Group II and IV. For the microbial composition analysis, pair-wise comparisons with Student’s T approach were used between experimental groups and the control group. Significant differences were found in *Ruminococcaceae* (*P* < 0.01) (Figure 2B) and *vadinBB60 group* (*P* < 0.05) (Figure 2C) between Group I and IV, *Ruminococcaceae* (*P* < 0.05) (Figure 2B) between Group I and IV.

**FIGURE 3:**
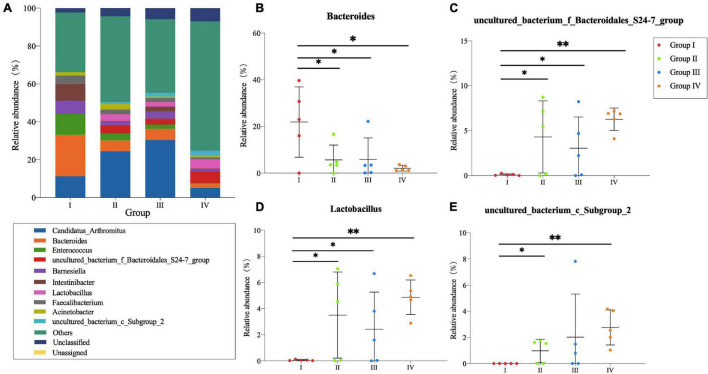
Community structure of bacteria at genus level in the ileum of *Zhijiang* ducks. Histogram **(A)** of the top 10 genera in each group. Significant difference relative abundance of *Bacteroides* **(B)**, *S24-7_group* **(C)**, *Lactobacillus* **(D)**, and *Subgroup_2* **(E)** at the genera level. (*P < 0.05 and ^**^P < 0.01 compared with the control group).

In the ileum (Figure 3A), *Candidatus Arthromitus* and *Bacteroides* were dominant genera in Groups I, II, and III which represented more than 30% of the genus type. However, they were detected in Group IV with low abundance. As for *Barnesiella*, *Intestinibacter*, and *Faecalibacterium*, higher abundance was observed in Group I. Additionally, the relative abundance of *S24-7 group*, *Lactobacillus*, and *Subgroup 2* in Group IV were higher compared with other groups. Through the Student’s *T*-test, there were significant differences of *Bacteroides*, *S24-7 group*, *Lactobacillus*, and *Subgroup 2* (*P* < 0.05) (Figures 3B–E) between Group I and test groups.

**FIGURE 4:**
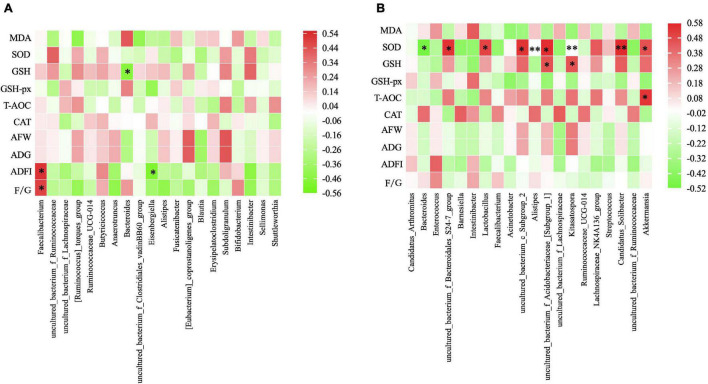
Heatmap of Spearman rank correlation between the cecal microbiota. **(A)** /ileum microbiota **(B)** and the measured parameters under different treatment. The genus with relative abundance in the top 10 are presented and the intensity of the colors performed the degree of association. Green, negative correlation; Red, positive correlation. AFW, average final weight; ADG, average daily weight gain; ADFI, average daily feed intake; F/G, feed to gain ratio. MDA, malonaldehyde; GSH, glutathione; SOD, activities of superoxide dismutase; GSH-Px, glutathione peroxidase; T-AOC, total antioxidant capacity; CAT, catalase. *P ≤ 0.05, ^**^P ≤ 0.01.

Furthermore, the correlation between the microbiota composition of ceacum/ileum and the indices of growth performance and antioxidative capacity were shown in Figure 4. After Spearman’s correlation analysis in ceacum, ADFI and F/G were found to have significant positive correlations with *Faecalibacterium* (*P* = 0.018 and *P* = 0.031), while negative correlations were examined between GSH and the *Bacteroides* (*P* = 0.039), and ADFI and the *Eisenbergiella* (*P* = 0.027). In ileum (Figure 4B), significantly positive results were observed between SOD and *S24-7 group* (*P* = 0.013), *Lactobacillus* (*P* = 0.024), *Subgroup 2* (*P* = 0.013), *Subgroup 1* (*P* = 0.010), *Kitasatospora* (*P* = 0.002), *Candidatus Solibacter* (*P* = 0.009), and *Akkermansia* (*P* = 0.040). Meanwhile, SOD was significantly negative in the *Bacteroides* (*P* = 0.024) and *Alistipes* (*P* = 0.009). In addition, significant positive correlations were existed between T-AOC and *Akkermansia* (*P* = 0.015),GSH and *Subgroup 1* (*P* = 0.042),and GSH and *Kitasatospora* (*P* = 0.042).

## Discussion

Several lines of studies demonstrated that *Bacillus* Probiotics could improve the growth performance of poultry (Jeong and Kim, 2014; Lee et al., 2015; Lin Y. et al., 2017). In the current study, we found that *Bacillus* probiotics supplementation could increase the average final weight and ADG by modulating antioxidative status and intestinal microflora. In fact, the antioxidant effect of *Bacillus* Probiotics has been verified in rats before (Paik et al., 2005). In our study, we found *Bacillus* probiotics have the efficacy of significantly decreasing the serum level of MDA and increasing the level of SOD and GSH. These results revealed that feeding ducks with *Bacillus* Probiotics can also improve the antioxidant status. Meanwhile, there were a higher relative proportion of the genera *Ruminococcaceae*, *vadinBB60 group, and S24-7 group* in the *Bacillus*-fed group in the cecal or ileal microbial community. Many species belonging to these genera are able to produce short-chain fatty acid (SCFA), especially butyrate (Hooda et al., 2012; Ridlon et al., 2015), which serves as a preferred energy source for enterocytes and a known regulator of cellular differentiation and proliferation within the intestinal mucosa (Sikandar et al., 2017; Bedford and Gong, 2018). This can contribute to improving the morphological development of the intestines and to reinforce the intestinal defense barrier, for instance, strengthening tight junctions, thereby promoting animal growth (Le Blay et al., 2000; Fukunaga et al., 2003; Bordin et al., 2004; Peng et al., 2007). Additionally, a significant increasing trend was also observed for *Lactobacillus* of the ileum in the *Bacillus*-treated group. This point deserves further investigation as it might be of interest, as *Lactobacillus* may provide nutrients to the host and help defend against opportunistic pathogens (Cross, 2002; LeBlanc et al., 2013). Interestingly, the *Bacteroides* genus was largely decreased with the presence of *Bacillus*, which conflicts with the findings of a very recent broiler study (Wang et al., 2017). However, studies using other species, such as swine, have shown that *Bacillus subtilis* supplementation decreased the copy numbers and percentage of *Bacteroidetes* while it increased the percentage of *Firmicutes* in the cecal contents (Cui et al., 2013). Similarly, a significant decrease trend was also revealed in *Bacteroidetes* of the ileum in our study. Given the fact that the decrease in *Bacteroidetes* and the increase in the *Firmicutes*/*Bacteroidetes* ratio are positively correlated with body mass index in humans (Koliada et al., 2017) and associated with an increase in ADG (Salaheen et al., 2017), our data revealed that *Bacillus* Probiotics promotes duck growth by improving the intestinal flora.

Nowadays, poultry feed with higher levels of NSP may reduce the digestibility of nutrients in their diets (Salim et al., 2010), which can lead to poorer growth and performance of birds (Annison and Choct, 1991). One solution to this issue accounting for feed cost and variability is to use exogenous enzymes (Woyengo et al., 2019), including NSPases. In our study, the addition of NSPases increased the average final weight and ADG of ducks, but insignificantly. A similar result was also obtained by Uthai et al. (2004). They found NSPase type mixture supplementation could improve the growth performances (average total weight gained, average daily gain, and average feed intake) of pigs also with non-statistically significant differences. Besides, Ao et al. (2010) reported that 2% NSPase supplementation to based diets for weaned piglets did not significantly improve performance. But interestingly, the average daily gain and gain/feed were increased 4.40 and 6.26% when fed with 1% NSPase supplementation. Variation in the results is likely associated with the content of substrates (Ao et al., 2010). However, a significant increase was found in the proportions of the *S24-7 group*, *Lactobacillus*, and *Subgroup 2*, while a significant decrease was found in the proportion of *Bacteroides* in the NSPase-treated group at the ileal level. Considering the results that the decrease in *Bacteroidetes* and the increase in *S24-7 group*, *Lactobacillus*, and *Subgroup 2* are positively correlated with SOD activity. Thus, ducks fed with NSPases showed improved growth performance, and increased antioxidant capacity might be linked to the microbial changes. In many studies, the change of intestinal microflora was beneficial for the health and welfare of poultry. Naumova et al. (2021) found that the beneficial effect on the production of ducks was associated with the changes in gut microbiota due to *Bacillus*-feed probiotic supplementation. Dietary probiotics can enhance growth performance by the regulation of intestinal microbial composition, the immune system, and maintenance of intestinal integrity and barrier function, as described by Tuohy et al. (2003). In our study, the major changes were found within the foregut area and the predominant role of *Lactobacillus* was enhanced. Lactobacillus consists of gram-positive and facultative anaerobes that produce lactic acid, which can create a low pH environment and inhibit the growth of pathogens (Rodriguez-Cabezas et al., 2010). In addition, Lactobacillus was proven to be of antioxidant activity (Lin and Chang, 2000), and having the capacity of scavenging free radicals to alleviate damages induced by oxidative stress (Xin et al., 2014). It indicated that the increase of the genus Lactobacillus probably enhances the antioxidant activity of duck. However, other bacterial species and their interaction (significantly positively associated with SOD) were still unclear.

In the present study, *Bacillus* probiotics and *NSPases* supplementation have been proven to be a potential method to improve the average final weight and ADG of ducks. Our findings are consistent with the result reported by Lin Q. et al. (2012), who demonstrated that *Bacillus* probiotics and NSPases showed obvious synergistic growth-promoting effects on yellow-feathered broilers. Most noteworthily, compared to the basal diet group, the diversity index of ACE and Chao increased when the treatment of NSPase or *Bacillus* Probiotics alone or combined, especially, the significantly increased (*P* < 0.05) of ACE and Chao revealed in group IV. The PCoA and NMDS results further indicated that the group of diet NSPases + *Bacillus* was far from the control group, which was consistent with the above ACE and Chao analysis results. Our results were consistent with Klein et al. (2015) and Liu et al. (2018). They reported that the beneficial effects may be observed from the use of multiple enzymes or mixed-use of Bacillus subtilis and photosynthetic bacteria. Moreover, a significant increase was found in proportions of *Ruminococcaceae*, *S24-7 group*, *Lactobacillus*, and *Subgroup 2*, and a significant decrease was found in the proportion of *Bacteroides* in NSPase + *Bacillus* Probiotics-treated group at the cecal or ileal level. An obvious change in ACE or Chao index would suggest the deep changes in microbial diversity. On one hand, NSPase might affect the intestinal microbiota by reducing the undigested substrates and some short-chain oligosaccharides with potential prebiotic effects were created (*in situ*) from cell wall NSP (Kiarie et al., 2013). On the other hand, *Bacillus* probiotics could influence the distribution and colonization of the innate microflora along the gastrointestinal tract, reduce the competition for nutrients between microbes and the host, promote the growth and proliferation of other good symbiotic bacteria (Grant et al., 2018). To summarize, it has been suggested that *Bacillus* Probiotics and NSPases have a significant synergistic effect on improving the intestinal microflora of ducks.

## Conclusion

Including *Bacillus* (25 mg/kg) and NSPases (150 mg/kg) in the diet could efficiently enhance growth performance via altering gut microbiota composition at the genera level and antioxidant indices of ducks. By comparison, the supplementation combining both *Bacillus* and NSPases showed the best effect for microbial abundances and diversities. Thus, using an additive of both *Bacillus* and NSPases could be recommended in diets during the early stage of *Zhijiang* ducks.

## Data Availability Statement

The data analyzed in this study is subject to the following licenses/restrictions: The data used to support the findings of this study are available from the corresponding author upon request. Requests to access these datasets should be directed to QL, linqian@caas.cn.

## Ethics Statement

The animal study was reviewed and approved by the experimental procedures of this study were approved by the Institutional Animal Care and Use Committee of Institute of Bast Fiber Crops, Chinese Academy of Agricultural Sciences.

## Author Contributions

HQ, QL, and QD conceived and designed the experiments. XW, YZW, TL, SP, and HZ prepared the samples. XW completed the serum indices measurement. YYW detected the growth performance index. YYW and QL performed the sequencing of the 16S rRNA gene. SP and SZ completed the data analysis. SP and QL wrote the manuscript. QL and JZ refined the article. All authors contributed to the article and approved the submitted version.

## Conflict of Interest

The authors declare that the research was conducted in the absence of any commercial or financial relationships that could be construed as a potential conflict of interest.

## Publisher’s Note

All claims expressed in this article are solely those of the authors and do not necessarily represent those of their affiliated organizations, or those of the publisher, the editors and the reviewers. Any product that may be evaluated in this article, or claim that may be made by its manufacturer, is not guaranteed or endorsed by the publisher.
